# Automatic Detection of Slow Conducting Channels during Substrate Ablation of Scar-Related Ventricular Arrhythmias

**DOI:** 10.1155/2020/4386841

**Published:** 2020-05-29

**Authors:** Alejandro Alcaine, Beatriz Jáuregui, David Soto-Iglesias, Juan Acosta, Diego Penela, Juan Fernández-Armenta, Markus Linhart, David Andreu, Lluís Mont, Pablo Laguna, Oscar Camara, Juan Pablo Martínez, Antonio Berruezo

**Affiliations:** ^1^CIBER en Bioingeniería, Biomateriales y Nanomedicina (CIBER-BBN), Zaragoza, Spain; ^2^BSICoS Group, Aragón Institute of Engineering Research (I3A), IIS Aragón, Universidad de Zaragoza, Zaragoza, Spain; ^3^BCN MedTech Unit, PhySense Group, Department of Information and Communication Technologies, Universitat Pompeu Fabra, Barcelona, Spain; ^4^Teknon Medical Center, Barcelona, Spain; ^5^Hospital Universitario Virgen del Rocío, Sevilla, Spain; ^6^Ospedale Guglielmo da Saliceto, Piacenza, Italy; ^7^Hospital Puerta del Mar, Cádiz, Spain; ^8^Arrhythmia Section, Cardiology, Hospital Universitari Doctor Josep Trueta, Girona, Spain; ^9^Boston Scientific, Madrid, Spain; ^10^Hospital Clínic, Universitat de Barcelona, Barcelona, Spain; ^11^Institut d'Investigacions Biomèdiques August Pi i Sunyer (IDIBAPS), Barcelona, Spain; ^12^CIBER en Enfermedades Cardiovasculares (CIBER-CV), Barcelona, Spain

## Abstract

**Background:**

Voltage mapping allows identifying the arrhythmogenic substrate during scar-related ventricular arrhythmia (VA) ablation procedures. Slow conducting channels (SCCs), defined by the presence of electrogram (EGM) signals with delayed components (EGM-DC), are responsible for sustaining VAs and constitute potential ablation targets. However, voltage mapping, as it is currently performed, is time-consuming, requiring a manual analysis of all EGMs to detect SCCs, and its accuracy is limited by electric far-field. We sought to evaluate an algorithm that automatically identifies EGM-DC, classifies mapping points, and creates new voltage maps, named “Slow Conducting Channel Maps” (SCC-Maps).

**Methods:**

Retrospective analysis of electroanatomic maps (EAM) from 20 patients (10 ischemic, 10 with arrhythmogenic right ventricular dysplasia/cardiomyopathy) was performed. EAM voltage maps were acquired during sinus rhythm and used for ablation. Preprocedural contrast-enhanced cardiac magnetic resonance (Ce-CMR) imaging was available for the ischemic population. Three mapping modalities were analysed: (i) EAM voltage maps using standard (EAM standard) or manual (EAM screening) thresholds for defining core and border zones; (ii) SCC-Maps derived from the use of the novel SCC-Mapping algorithm that automatically identify EGM-DCs measuring the voltage of the local component; and (iii) Ce-CMR maps (when available). The ability of each mapping modality in identifying SCCs and their agreement was evaluated.

**Results:**

SCC-Maps and EAM screening identified a greater number of SCC entrances than EAM standard (3.45 ± 1.61 and 2.95 ± 2.31, resp., vs. 1.05 ± 1.10; *p* < 0.01). SCC-Maps and EAM screening highly correlate with Ce-CMR maps in the ischemic population when compared to EAM standard (Lin's correlation = 0.628 and 0.679, resp., vs. 0.212, *p* < 0.01).

**Conclusion:**

The SCC-Mapping algorithm allows an operator-independent analysis of EGM signals showing better identification of the arrhythmogenic substrate characteristics when compared to standard voltage EAM.

## 1. Introduction

Voltage mapping allows the characterization of myocardial scar, being a useful tool for ablation of scar-related ventricular arrhythmias (VA) [1–3]. Small bundles of viable cardiac myocytes within the scar create slow conducting channels (SCCs) that are responsible of the formation of reentrant circuits promoting VA [3–9]. Computer software for electroanatomical mapping (EAM) allows the quantification of local electrogram (EGM) voltages as the peak-to-peak differences of each bipolar EGM signal [10]. However, far-field activity from surrounding healthy tissue can result in underestimation of the scar area and may lead to a worse definition of EGM signals with delayed components (EGM-DC), thus masking the presence of SCCs.

The “scar dechanneling” technique has been introduced as a substrate ablation strategy for scar-related VAs, either for ischemic or nonischemic cardiomyopathy [8, 9, 11]. Briefly, this technique is based on bipolar voltage mapping of the scar during sinus rhythm (SR), analysis of EGMs to identify SCCs, and ablation of all the identified SCC entrances. Outcomes of the “scar dechanneling” technique depend on the correct identification and elimination of all present SCCs [9]. This can be a time-consuming and skill-demanding task, being subject to significant interoperator variability. We hypothesize that an automatic system able to identify EGM-DC within the substrate could simplify and standardize VA ablation procedures.

In this study, we present and evaluate the performance of a novel algorithm for automatic EGM analysis so called “Slow Conducting Channel Mapping” algorithm, or “SCC-Mapping.” This algorithm dichotomizes normal from abnormal bipolar EGMs, automatically identifying the presence of EGM-DC within the substrate. By measuring the bipolar voltage belonging to the local-field component, the SCC-Mapping algorithm may obtain more accurate bipolar voltage maps. Thus, a better scar characterization could help to guide scar-related VA ablation procedures.

## 2. Methods

### 2.1. Patient Sample

Twenty patients (fifteen males) with VA who underwent catheter-based radiofrequency ablation were included in the study. Ten patients (nine males) had ischemic cardiomyopathy. Ten (six males) fulfilled Task Force criteria for arrhythmogenic right ventricular dysplasia/cardiomyopathy (ARVD/C). Ischemic patients were selected from our database of VA substrate ablation as consecutive patients having preprocedural contrast-enhanced cardiac magnetic resonance (Ce-CMR) imaging study. Basal characteristics of the population are summarized in Table 1. The study complied with the Declaration of Helsinki, and the local ethics committee approved the study protocol. All participants included in the study provided informed written consent.

### 2.2. Mapping and Ablation Procedure

Electroanatomical maps (EAM) were obtained with the CARTO3® navigation system (Biosense Webster, Inc., Diamond Bar, CA, USA) using a 3.5 mm irrigated-tip ThermoCool® SmartTouch® catheter (Biosense Webster, Inc., Diamond Bar, CA, USA) for mapping and ablation. Bipolar electrograms were filtered from 30 to 250 Hz. The 12-lead surface electrocardiogram (ECG) and EGM signals from the mapping catheter were displayed and stored for prospective analysis. Endocardial EAM maps were acquired for all patients belonging to the ischemic subpopulation and in seven of the patients from the ARVD/C subpopulation, the rest of EAM maps of the ARVD/C subpopulation were obtained from the epicardium.

Ablation was performed under conscious sedation or general anaesthesia when epicardial access was required or anticipated. Bipolar voltage maps were obtained during SR and scar was identified using standard voltage thresholds defining scar core zone (CZ) (<0.5 mV), border zone (BZ) (<1.5 mV), and healthy tissue (≥1.5 mV). The “scar dechanneling” ablation technique was used for identification and ablation of SCC entrances, thus isolating the VA isthmuses [9]. Identification of SCC was performed manually by the EAM navigation system operator. After ablation of all the SCC entrances, a remap procedure was performed to detect any residual SCC and ablate them if needed. When finished, a programmed stimulation protocol was performed, remapping again the substrate in case any sustained VAs were found inducible, until noninducibility was achieved. Ablation was performed in temperature-controlled mode with 45°C temperature and 50 W power limit at 26 mL/min irrigation rate (40 W and 17 mL/min at epicardium).

### 2.3. Ce-CMR Acquisition and Processing

A preprocedural late gadolinium Ce-CMR was acquired in all ischemic cases and used to localize the arrhythmogenic substrate [12]. The preprocedural Ce-CMR studies were obtained using a 3T scanner (MAGNETOM® Trio®, Siemens Healthcare, Erlangen, Germany). Contrast-enhanced images were acquired 10 minutes after bolus injection of 0.2 mmol/kg Gadobutrol (Gadovist®, Bayer Hispania, Barcelona, Spain) using a commercially available, free-breathing, ECG-gated, navigator-gated, 3D inversion-recovery, gradient-echo technique.

Ce-CMR images were analysed as previously described [12]. Briefly, a full left ventricular (LV) volume was reconstructed in the axial orientation, and the resulting images were processed with the commercially available ADAS-3D™ software (Galgo Medical, Barcelona, Spain). Ten concentric surface layers (from 10% to 90%) were created automatically from endo- to epicardium of the LV wall thickness. A 3D shell was obtained for each layer. Pixel signal intensity (PSI) maps based on Ce-CMR images were projected to each shell, following a trilinear interpolation algorithm, and colour-coded. To identify the scar areas, a PSI-based algorithm was applied to characterize the hyperenhanced area as CZ, BZ or healthy tissue using 40% ± 5% and 60% ± 5% of the maximum intensity as thresholds [12]. The BZ channels (i.e., SCCs) were defined by the ADAS-3D™ software as continuous 3D corridors (across all the Ce-CMR layers) of BZ (with the specified PSI threshold) surrounded by scar core/mitral annulus [12].

### 2.4. The “Slow Conducting Channel Mapping” (“SCC-Mapping”) Algorithm

The SCC-Mapping algorithm is based on an EGM detector and delineator algorithm previously developed by our team [13]. This detector automatically identifies and delineates the onset and end landmarks of the bipolar EGM signal using the QRS complex of the 12-lead surface ECG as the reference searching window, a method that has been already validated for activation mapping of focal VA [13, 14]. The entire processing algorithm was implemented in MATLAB® (MATLAB R2016a, MathWorks, Inc., Natick, MA, USA). The results were obtained offline; therefore, ablation outcomes were independent of the presented results.

**Figure 1:**
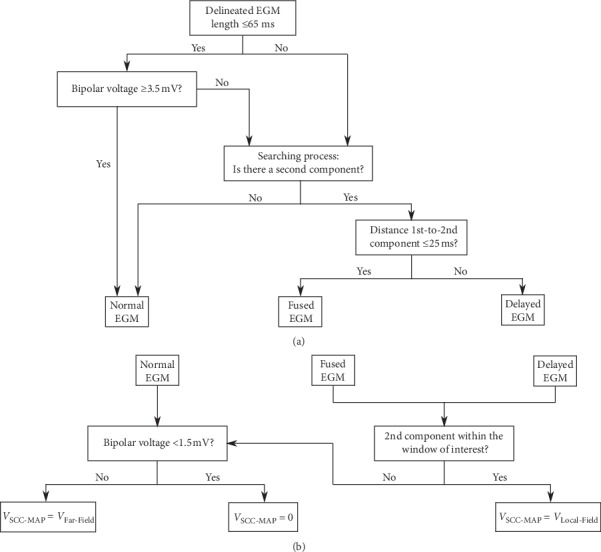
The “Slow Conducting Channel Mapping Algorithm.” (a) Decision tree for electrogram (EGM) signals with delayed components (EGM-DC) searching protocol and (b) algorithm for the reconstruction of “Slow Conducting Channel Maps” (SCC-Maps) on patient's 3D anatomical map. VSCC-Map: bipolar voltage projected on patient's 3D anatomical map.

Starting from an initial delineation of the mapping point EGM signal using our EGM detector/delineator algorithm [13], the SCC-Mapping algorithm uses a decision tree illustrated in Figure 1(a). This decision tree is based on two main characteristics of the bipolar EGM signal: the delineated length and the bipolar voltage.

For short-duration EGMs (<65 ms, based on [15]), normal mapping points are distinguished from those candidates to be an EGM-DC by the measured bipolar voltage. Therefore, those mapping points showing a bipolar voltage ≥3.5 mV were considered normal EGMs [15], whereas the rest were candidates for being classified as EGM-DC or remained as normal EGMs. Long-duration EGMs (>65 ms) were always considered potential candidates for EGM-DC, regardless of their voltage value.

In order to label an EGM-DC candidate with true delayed (d-EGM) or fused (f-EGM) components, the algorithm searched for the existence of a second EGM component based on the EGM detector/delineator algorithm [13]. If a second EGM component was found, the time distance between the main deflection of far- and local-field component (i.e., the first and the second component) dichotomized between d-EGMs and f-EGMs (see Figure 1(a)). The cut-off threshold was set to 25 ms as a trade-off for good identification of f-EGM and d-EGM signals. f-EGMs were ablation targets according to the “scar dechanneling” technique, as they constitute the typical pattern at SCC entrances [9].

The outcome of the algorithm is the identification label on the type of EGM assigned to each mapping point. These labels were then colour-coded and integrated into the 3D EAM with the following criteria: small white spheres for normal EGM mapping points; blue big spheres for d-EGM mapping points, and black big spheres for f-EGMs mapping points.

### 2.5. Construction of SCC-Maps

The SCC-Mapping algorithm identifies the existence of potential EGM-DC, allowing to measure the bipolar amplitude of far- and local-field components individually. The projection of these voltages on a 3D SCC-Map was performed using an additional decision tree (Figure 1(b)) with two branches: one for single-component EGMs and another for EGM-DC (either d-EGMs or f-EGMs). The single-component EGM branch considered any mapping point <1.5 mV as far-field remote signals measured within the dense scar coming from the surrounding healthy myocardium, automatically setting its bipolar voltage to zero. The double-component EGM branch projects on the 3D SCC-Map the bipolar voltage of the local-field component only if it is included within a window of interest. This window of interest was defined between the 5th and 95th percentiles of the onsets and ends of all identified local-field components, respectively. If the local-field component of an EGM-DC mapping point did not meet this criterion, then the same criterion as for single-component EGM mapping points was applied. Additionally, the algorithm includes a spatial coherence protection. This protection checks, for close EGM-DC mapping points (distance < 6 mm), if the local-field components are similar in activation time and shape. When these criteria are met, only the highest bipolar value is represented in the 3D SCC-Map.

### 2.6. SCC Detection Agreement Evaluation

In this study, three mapping modalities were considered: (i) EAM voltage maps, (ii) SCC-Maps derived from the SCC-Mapping Algorithm; and (iii) Ce-CMR PSI maps (when available). An expert operator visually evaluated the ability of each mapping modality to identify SCC entrances from both the 3D coloured map and the acquired EGM signals.

#### 2.6.1. Identification of SCC Entrances Derived from the 3D Coloured Map

For the EAM voltage maps, SCC entrance identification was performed using (1) the standard thresholds that define the presence of scar CZ and BZ tissue (named “EAM standard”); and (2) using a manual voltage screening process that dynamically modifies the standard thresholds for BZ and CZ definition in order to enhance the presence of SCCs (named “EAM screening”) [3, 16]. For the SCC-Maps, this process was done directly from the coloured map. It should be noted that, due to the more precise bipolar voltage measurement method of SCC-Maps, the CZ tissue threshold was set to ≤0.1 mV, yielding a higher range of bipolar voltage measurements (shown and discussed in the following sections). Therefore, the voltage screening process was not necessary for SCC-Maps. For the Ce-CMR PSI maps, the threshold definitions for tissue heterogeneity identification (i.e., BZ tissue that conforms SCCs) were those explained in the corresponding section.

#### 2.6.2. Identification of SCC Entrances Derived from the Analysed EGM Signals

This process was done by manual inspection of the presence or absence of f-EGMs in the identified (labelled) mapping points close to the BZ area. Therefore, this evaluation could only be done for EAM voltage maps and SCC-Maps.

### 2.7. Statistical Analysis

Continuous data are shown in mean ± standard deviation, unless otherwise indicated. Categorical data are shown as percentages. Comparison between different populations was given by the Wilcoxon–Mann–Whitney test or by the Fisher exact test when appropriate. For evaluation of the agreement in the SCC entrances identification among the different mapping modalities, Wilcoxon–Mann–Whitney test, Lin's concordance correlation factor “*ρ*” [17], and Bland–Altman plot analysis [18] were used. A *p*-value of ≤0.05 was considered as a cut-off value for statistical significance. Statistics were obtained using the MATLAB statistics toolbox (MATLAB R2016a, MathWorks, Inc., Natick, MA, USA).

## 3. Results

### 3.1. Population Characteristics

Twenty patients were included in the study. 75% were male, and mean age was 57 ± 15 years. Mean LV ejection fraction was 44 ± 16%, with no significant differences between ischemic patients and those with ARVD/C. Table 1 summarizes the baseline characteristics of the study population.

### 3.2. SCC Detection Agreement between EAM and SCC-Mapping

**Figure 2:**
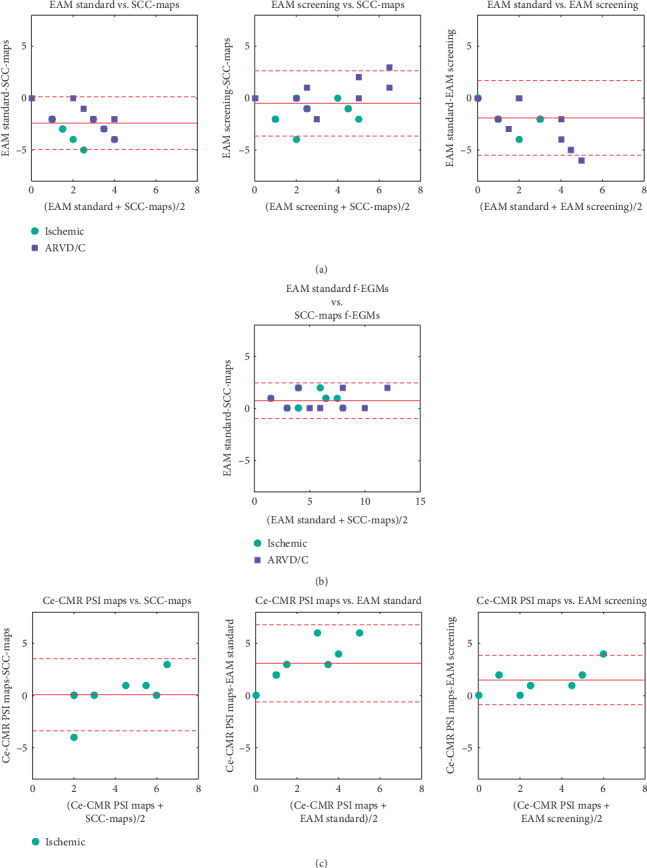
Bland–Altman plots for assessing the agreement in the identification of slow conducting channel (SCC) entrances (a) from the colour-coded 3D maps between the different mapping modalities: electroanatomical mapping (EAM) system maps with standard voltage thresholds (EAM standard), EAM maps with voltage screening (EAM screening), and “Slow Conducting Channel Maps” (SCC-Maps). (b) From the analysis of the presence of fused electrograms (f-EGM) components between EAM standard maps and SCC-Maps and (c) from the colour-coded 3D map between the different mapping modalities and the pixel signal intensity (PSI) maps derived from contrast-enhanced cardiac magnetic resonance (Ce-CMR) imaging in the ischemic population. Red solid line indicates mean and red dashed lines indicate mean ± 2 standard deviations of the difference in the number of identified SCC entrances. ARVDC: arrhythmogenic right ventricular dysplasia/cardiomyopathy.

Table 2 and Figure 2(a) show the agreement in the number of SCC entrances identified from the 3D coloured maps between all the studied mapping modalities. EAM voltage maps with standard thresholds (“EAM standard”) presented a significant lower number of SCC entrances than EAM voltage maps with manual voltage screening (“EAM screening”) (*p* < 0.01, 0.04,  and 0.03 for the entire population, ischemic, and ARVD/C, resp.). Additionally, EAM standard maps also had less SCC entrances than SCC-Maps (*p* < 0.01, <0.01,  and 0.02 for the entire population, ischemic, and ARVD/C, resp.). However, there were no significant differences in the number of identified SCC entrances between EAM screening maps and SCC-Maps (*p*=0.29, 0.10,  and 0.87 for the entire population, ischemic, and ARVD/C, resp.). Lin's concordance correlation factor analysis shown in Supplementary Table 1 supports these findings, showing a higher concordance between EAM screening and SCC-Maps (*ρ* = 0.665, 0.528, and 0.877 for the entire population, ischemic, and ARVD/C, resp.) than EAM standard maps.

The Bland–Altman analysis shown in Figure 2(a) illustrates the agreement in SCC entrance identification among the different studied mapping modalities and populations. There was a low bias in the number of SCC entrances identified between SCC-Maps and EAM screening maps, with a small trend towards a SCC subidentification of SCC-Maps compared with EAM screening maps (Pearson's *R* = 0.48, *p*=0.033), which is in concordance with the findings of Table 2.

Table 3 and Figure 2(b) list the agreement in the number of SCC entrances identified from the f-EGM points between mapping modalities. No significant differences were found between EAM standard maps and SCC-Maps, confirmed by the Bland–Altman analysis shown in Figure 2(b) and the high correlation shown in Supplementary Table 2 (*ρ* = 0.918, 0.871, and 0.936 for the entire population, ischemic, and ARVD/C, resp.).

**Figure 3:**
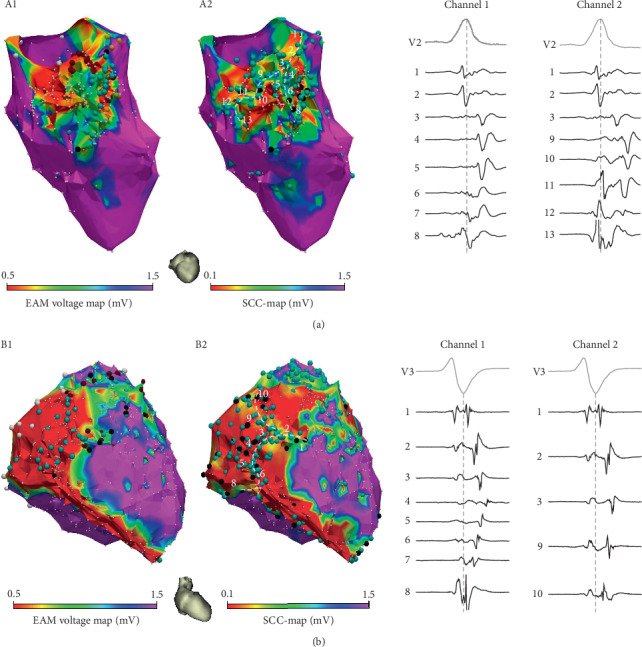
Examples of slow conducting channel (SCC) identification from the automatic mapping point labelling on “Slow Conducting Channel Maps” (SCC-Maps). (a) Endocardial electroanatomical map (EAM) of an ischemic patient showing two SCCs identified on SCC-Map. (b) Epicardial EAM from an arrhythmogenic right ventricular dysplasia/cardiomyopathy patient showing two SCCs identified on SCC-Map.

Figure 3 illustrates two examples of the electrical propagation sequences along SCCs identified from the automatically labelled mapping points on SCC-Maps compared to the SCC manual identification performed on EAM maps where the identification of these SCCs requires extensive operator analysis.

### 3.3. SCC Detection Agreement with Ce-CMR

**Figure 4:**
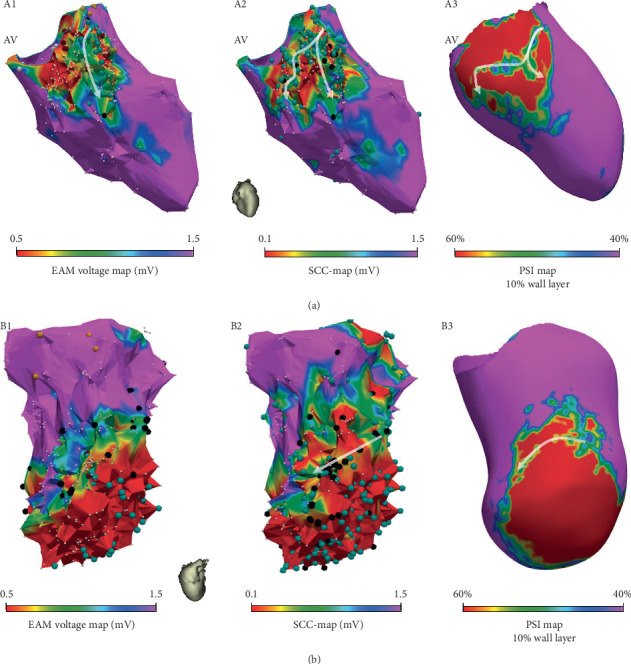
Agreement between electroanatomical mapping (EAM) voltage maps and “Slow Conducting Channel Maps” (SCC-Maps) against pixel signal intensity (PSI) maps derived from contrast-enhanced cardiac magnetic resonance (Ce-CMR) imaging. A1 and B1 show the electroanatomical mapping (EAM) voltage maps obtained with the EAM system from two different patients. A2 and B2 show the corresponding SCC-Map and A3 and B3 show the acquired Ce-CMR PSI map. AV: aortic valve.

The number of SCC entrances identified in EAM standard was significantly lower compared with Ce-CMR PSI maps, as shown in Table 2. However, no significant differences were found between EAM screening and SCC-Maps versus Ce-CMR PSI maps (*p*=0.202 and *p*=1.0, resp.). Nevertheless, Bland–Altman plot analysis reveals a tendency of these three mapping modalities towards an underestimation of the number of SCC entrances compared with Ce-CMR PSI maps (Pearson's *R* = 0.63, *p*=0.049; *R* = 0.85, *p*=0.002; and *R* = 0.61, *p*=0.05 for the comparisons between SCC detection on SCC-Maps, EAM standard, and EAM screening against Ce-CMR PSI maps, resp., Figure 2(c)). Moreover, Supplementary Table 1 confirms the high agreement of EAM screening and SCC-maps with Ce-CMR PSI maps compared with EAM standard (*ρ* = 0.679 and *ρ* = 0.628, resp., vs. *ρ* = 0.212, *p* < 0.01).  Figure 4 shows two examples where SCC-Maps had higher agreement with Ce-CMR PSI maps in identifying SCC, as compared to EAM standard maps.

### 3.4. Effect of Selective Bipolar Voltage Measurement

**Figure 5:**
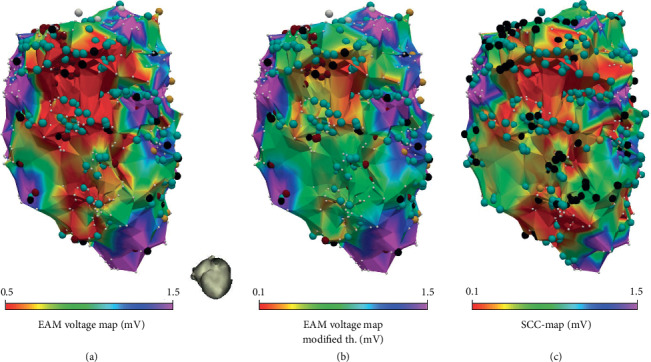
Endocardial substrate map from patient with myocardial infarction. (c) illustrates the richest scar details shown by the “Slow Conducting Channel Map” (SCC-Map) compared with electroanatomical mapping (EAM) voltage maps using the standard voltage thresholds (a) and using modified voltage thresholds (b).

The SCC-Mapping algorithm was capable of detecting EGM-DC providing the bipolar voltage of the local component, thus obtaining accurate voltage maps (SCC-Maps). This more selective approach enlarged the voltage range displayed and thus improved the degree of detail of the arrhythmogenic substrate when compared with standard EAM voltage maps.  Figure 5(b) illustrates the loss of scar details in EAM voltage maps when compared to SCC-Maps, which display a higher range of voltage measurements. Additionally, as depicted in Figure 4, SCC-Maps matched better with information obtained from Ce-CMR PSI maps in the ischemic population than EAM standard maps.

## 4. Discussion

### 4.1. Reviewing Current Bipolar Voltage Mapping

EAM systems are useful tools to map scar-related VAs, since they allow calculating the peak-to-peak local EGM signal amplitude and representing this value, colour-coded, on the cardiac anatomy, thus helping to identify and characterize the scar [3, 5, 6, 8–10, 19, 20]. However, myocardial scars are often surrounded by a considerable amount of healthy tissue, which may lead to local EGMs being masked by the presence of far-field signals. Therefore, regular voltage mapping with standard thresholds may underestimate of the scar size and lose significant scar details. This increases the likelihood of missing SCCs within the substrate and the need of extensive operator analysis.

**Figure 6:**
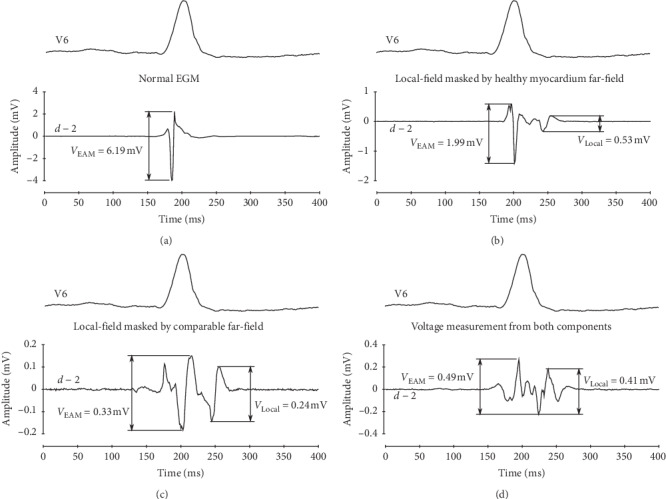
Examples of (a) normal electrogram (EGM) bipolar voltage measurement by the electroanatomical mapping (EAM) system and (b–d) different bipolar EGM-DC signals with incorrect bipolar voltage measurement by the EAM system: (b) local-field component masked by high-amplitude far-field component. (c-d) Comparable amplitude of far-field and local-field components.

Examples of this phenomenon are illustrated in Figure 6. In normal EGMs, the voltage map reflects the peak-to-peak voltage (a); however, when healthy myocardium EGM (i.e., far-field) has a higher amplitude than the late potential (i.e., local-field), the voltage map reflects the far-field, high-amplitude component voltage (b). Moreover, when the far- and local-field components show comparable amplitudes, voltage map may reflect the peak-to-peak amplitude of either the local or the far-field component (c), or a mix of both (d). These examples show the need of a more selective approach to measure bipolar voltage for substrate mapping of scar-related VAs.

### 4.2. Main Findings

The present study evaluates a novel automatic EGM signal analysis algorithm aiming to improve the accuracy of current voltage mapping obtained with EAM systems. This algorithm allows obtaining voltage maps with higher voltage range, hence depicting more detailed scar characteristics, which may be useful to identify VA isthmuses during ablation procedures. The main findings of the study are as follows: (1) the proposed SCC-Mapping algorithm automatically identified SCC entrances at the same level as manual EAM voltage screening; (2) the SCC-Mapping algorithm provided SCC-Maps that match Ce-CMR PSI maps better than current EAM voltage maps; and (3) the SCC-Mapping algorithm improves the definition of the scar CZ and BZ areas by allowing a higher voltage range.

### 4.3. SCC Detection and Agreement between Mapping Modalities

SCC-Maps were highly correlated with EAM standard maps when these were obtained after a manual voltage screening process (EAM screening), whereas raw EAM standard maps (EAM standard) correlated worse and identified a significant lower number of SCC entrances. The algorithm's accuracy is illustrated by the fact that SCC-Maps can display a detailed scar without the need of manual voltage screening, with good agreement with EAM after manual labelling of the mapping points corresponding to SCC entrances (i.e., those showing f-EGM signals).

Figure 3 shows ischemic and ARVD/C patient examples where two different SCCs can be found. Both examples illustrate the superiority of SCC-Maps over EAM standard maps, allowing the identification of those signal corridors by direct inspection of the colour map. The EAM standard map of Figure 3(a) (panel A1) depicts a dense scar in the area where a second SCC can be found following the activation sequence of the d-EGMs. This SCC can be easily identified by the colour map and automatic labelling in Figure 3(a) (panel A2). Similarly, the EAM standard map of Figure 3(b) (panel B1) does not allow to identify those SCCs that can be seen in the SCC-Map of Figure 3(b) (panel B2). Moreover, SCC-Maps and EAM-screening correlate better than EAM-standard maps with Ce-CMR PSI maps. These examples illustrate the need of manual analysis of the EGM signals by the system operator in order to identify all the possible SCCs present in the substrate (i.e., using techniques like manual voltage screening and individual EGM labelling). This procedure can be guided and shortened by the proposed automatic SCC-Mapping algorithm. Moreover, automatic and objective identification becomes mandatory when using the increasingly popular multielectrode mapping (MEM) catheters where tenths of simultaneous signals per beat can be acquired.

### 4.4. Voltage Thresholds for Scar Definition

The proposed SCC-mapping algorithm provides a more precise quantification of the local-field voltage. This aspect allows changing the threshold definition for CZ tissue without losing scar information, thus improving the ability to detect of SCC (Figure 4). This effect is comparable to the one obtained with current MEM catheters [21], but using a regular electrode-size catheter in conjunction with an automatic algorithm to distinguish the far- and local-field components of the measured EGMs. In contrast, MEM catheters still need extensive operator analysis in order to identify and/or enhance the presence of SCCs. This fact could be mitigated if high-density mapping is combined with an automatic algorithm as the presented in this work.

Figure 5 illustrates the loss of scar definition when the modified voltage threshold for BZ and CZ tissue is used on EAM voltage maps. The higher voltage range displayed by SCC-Maps also facilitates the voltage screening process for SCC identification. However, although SCC-Maps and EAM screening maps provide similar insights, the former were obtained without any manual intervention, thus being operator independent. Additionally, as shown in Figures 3–5, it can be observed that SCC-Maps provided a better defined scar delineation than current EAM voltage mapping.

The proposed algorithm was evaluated when using the “scar dechanneling” ablation technique. However, the fact that a higher voltage range can be described with SCC-Maps allows a more detailed scar characterization, which suggests that the algorithm can also be useful for other ablation approaches [5, 22–25]. Eventually, the SCC-Mapping algorithm could effectively improve the guidance of pacing/entrainment manoeuvres for VA isthmus identification based on the tagged data and SCC-Map information.

### 4.5. Study Limitations

The main limitation of this study was the relatively small sample size. Comparison of EAM maps against Ce-CMR PSI maps was only possible in the ischemic population due to the presence of implantable devices in the ARVD/C population. On the other hand, the number of available patients with detailed EAM and quality Ce-CMR data has reduced lately, as detailed EAM acquisition is a time-consuming and highly operator-dependent task, while Ce-CMR-guided catheter substrate ablation has gained more interest [26]. Despite this trend, the endpoints for determining the ablation targets are still based on EAM findings where the presented algorithm can play an important and complementary role. Also, this algorithm paves the way for better integration of Ce-CMR and EAM data to improve scar-related VT ablation procedures.

Additionally, the algorithm was designed and tested using data from substrate-based VA ablation during SR. Hence, no data from VA mapping was used or analysed with this algorithm and therefore, other possible VA isthmuses were not explored with this algorithm [27].

In this work, EAMs were acquired using a standard 3.5 mm irrigated-tip mapping catheter, which has a longer interelectrode distance as compared with high-density MEM catheters that can better discriminate local- from far-field components [21]. However, manual annotation of multiple simultaneous signals obtained with MEM is a nonaffordable task, for which an automatic approach (like the SCC-Mapping algorithm) becomes necessary.

## 5. Conclusions

The proposed automatic analysis of EGM signals using the “Slow Conducting Channel Mapping Algorithm” improves the accuracy of bipolar voltage measurements within the scar area, achieving a more detailed tissue characterization and being an operator-independent tool for accurate identification of SCCs. This last feature encourages the use of the algorithm together with EAM navigation systems as a reproducible approach for guiding VA ablation procedures in daily practice.

## Figures and Tables

**Table 1:** Baseline characteristics of the study population.

	Entire population (*n* = 20)	Ischemic (*n* = 10)	ARVD/C (*n* = 10)	*p*-value^*∗*^
Age (years)	57 ± 15	69 ± 8	45 ± 9	<0.001
Sex (male)	15 (75%)	9 (90%)	6 (60%)	0.303
Hypertension (*n*)	7 (35%)	6 (60%)	1 (10%)	0.057
Dyslipidemia (*n*)	8 (40%)	7 (70%)	1 (10%)	0.020
LVEF (%)	44 ± 16	35 ± 20	49 ± 14	0.193
EAM points (*n*)	532 ± 212	438 ± 208	626 ± 180	0.076

Values are given as mean ± standard deviation or *n* (%). ^*∗*^*p*-value refers to the comparison between ischemic and ARVD/C populations. ARVD/C: arrhythmogenic right ventricular dysplasia/cardiomyopathy; LVEF: left ventricular ejection fraction; and EAM: electroanatomical mapping.

**Table 2:** Analysis of colour-coded maps. Number of SCC entrances identified per patient and agreement between mapping modalities.

	EAM standard	EAM screening	SCC-Map	Ce-CMR PSI maps	*p*-value^*∗*^	*p*-value^†^	*p*-value^‡^
Entire population (*n* = 20)	1.05 ± 1.10	2.95 ± 2.31	3.45 ± 1.61	N/A	<0.01	<0.01	0.29
Ischemic (*n* = 10)	0.60 ± 1.00	2.20 ± 1.75	3.60 ± 1.43	3.70 ± 2.45	0.04	<0.01	0.10
ARVD/C (*n* = 10)	1.50 ± 1.08	3.70 ± 2.63	3.30 ± 1.83	N/A	0.03	0.02	0.87

Number of SCC entrances per patient are given as mean ± standard deviation. ^*∗*^Differences between EAM standard and EAM screening. ^†^Differences between EAM standard and SCC-Maps. ^‡^Differences between EAM screening and SCC-Maps. ARVD/C: arrhythmogenic right ventricular dysplasia/cardiomyopathy; Ce-CMR: contrast-enhanced cardiac magnetic resonance; EAM: electroanatomical mapping; N/A: not applicable; PSI: pixel signal intensity; and SCC: slow conducting channel.

**Table 3:** Analysis of EGM-DC and identification of f-EGMs. Number of SCC entrances identified per patient and agreement between mapping modalities.

	EAM maps	SCC-Map	*p*-value
Entire population (*n* = 20)	6.10 ± 2. 81	5.35 ± 2.70	0.430
Ischemic (*n* = 10)	5.50 ± 2.17	4.70 ± 2.11	0.422
ARVD/C (*n* = 10)	6.70 ± 3.34	6.00 ± 3.16	0.790

Number of SCC entrances per patient are given as mean ± standard deviation. ARVD/C: arrhythmogenic right ventricular dysplasia/cardiomyopathy; EAM: electroanatomical mapping; EGM-DC: electrograms with delayed components; f-EGM: fused electrograms; and SCC: slow conducting channel.

## Data Availability

The electroanatomical mapping and image data used to support the findings of this study are restricted by the Hospital Clínic Local Ethical Committee in order to protect patient privacy. Data are available from Dr. Lluís Mont, PhD, Arrhythmias Unit, Hospital Clinic, Carrer de Villarroel, 170, 08036 Barcelona, Spain, for researchers who meet the criteria for access to confidential data.

## Supplementary Materials

Supplementary Materials Supplementary Table 1: Lin's concordance correlation factor on the number of SCC entrances identified between mapping modalities (rows against columns) from the analysis of the colour-coded maps. Supplementary Table 2: Lin's concordance correlation factor on the number of SCC entrances identified between EAM standard maps and SCC-Maps from the analysis of EGM-DC and identification of f-EGMs.

## References

[1] Callans D. J., Ren J.-F., Michele J., Marchlinski F. E., Dillon S. M. (1999). Electroanatomic left ventricular mapping in the porcine model of healed anterior myocardial infarction. Correlation with intracardiac echocardiography and pathological analysis. *Circulation*.

[2] Reddy V. Y., Wrobleski D., Houghtaling C., Josephson M. E., Ruskin J. N. (2003). Combined epicardial and endocardial electroanatomic mapping in a porcine model of healed myocardial infarction. *Circulation*.

[3] Arenal A., del Castillo S., Gonzalez-Torrecilla E. (2004). Tachycardia-related channel in the scar tissue in patients with sustained monomorphic ventricular tachycardias: influence of the voltage scar definition. *Circulation*.

[4] de Bakker J. M., van Capelle F. J., Janse M. J. (1988). Reentry as a cause of ventricular tachycardia in patients with chronic ischemic heart disease: electrophysiologic and anatomic correlation. *Circulation*.

[5] Arenal A., Glez-Torrecilla E., Ortiz M. (2003). Ablation of electrograms with an isolated, delayed component as treatment of unmappable monomorphic ventricular tachycardias in patients with structural heart disease. *Journal of the American College of Cardiology*.

[6] Cronin E. M., Al-Khatib S. M., Anter E. (2019). 2019 HRS/EHRA/APHRS/LAHRS expert consensus statement on catheter ablation of ventricular arrhythmias. *Europace*.

[7] Wylie J. V., Smith T. W., Josephson M. E., Shenasa M., Hindricks G., Borggrefe M., Breithardt G. (2009). Substrate mapping for ablation of ventricular tachycardia in coronary artery disease. *Cardiac Mapping*.

[8] Berruezo A., Fernández-Armenta J., Mont L. (2012). Combined endocardial and epicardial catheter ablation in arrhythmogenic right ventricular dysplasia incorporating scar dechanneling technique. *Circulation: Arrhythmia and Electrophysiology*.

[9] Berruezo A., Fernández-Armenta J., Andreu D. (2015). Scar dechanneling: new method for scar-related left ventricular tachycardia substrate ablation. *Circulation: Arrhythmia and Electrophysiology*.

[10] Issa Z. F., Miller J. M., Zipes D. P. (2012). *Clinical Arrhythmology and Electrophysiology: A Companion to Braunwald’s Heart Disease*.

[11] Fernández-Armenta J., Andreu D., Penela D. (2014). Sinus rhythm detection of conducting channels and ventricular tachycardia isthmus in arrhythmogenic right ventricular cardiomyopathy. *Heart Rhythm*.

[12] Andreu D., Ortiz-Pérez J. T., Fernández-Armenta J. (2015). 3D delayed-enhanced magnetic resonance sequences improve conducting channel delineation prior to ventricular tachycardia ablation. *EP Europace*.

[13] Alcaine A., Soto-Iglesias D., Calvo M. (2014). A wavelet-based electrogram onset delineator for automatic ventricular activation mapping. *IEEE Transactions on Biomedical Engineering*.

[14] Alcaine A., Soto-Iglesias D., Acosta J. (2018). Automatic activation mapping and origin identification of idiopathic outflow tract ventricular arrhythmias. *Journal of Electrocardiology*.

[15] Cassidy D. M., Vassallo J. A., Marchlinski F. E., Buxton A. E., Untereker W. J., Josephson M. E. (1984). Endocardial mapping in humans in sinus rhythm with normal left ventricles: activation patterns and characteristics of electrograms. *Circulation*.

[16] Mountantonakis S. E., Park R. E., Frankel D. S. (2013). Relationship between voltage map “channels” and the location of critical isthmus sites in patients with post-infarction cardiomyopathy and ventricular tachycardia. *Journal of the American College of Cardiology*.

[17] Lin L., Hedayat A. S., Sinha B., Yang M. (2002). Statistical methods in assessing agreement: models, issues, and tools. *Journal of the American Statistical Association*.

[18] Altman D. G., Bland J. M. (1983). Measurement in medicine: the analysis of method comparison studies. *The Statistician*.

[19] Marchlinski F. E., Callans D. J., Gottlieb C. D., Zado E. (2000). Linear ablation lesions for control of unmappable ventricular tachycardia in patients with ischemic and nonischemic cardiomyopathy. *Circulation*.

[20] Fernández-Armenta J., Berruezo A., Andreu D. (2013). Three-dimensional architecture of scar and conducting channels based on high resolution ce-CMR: insights for ventricular tachycardia ablation. *Circulation: Arrhythmia and Electrophysiology*.

[21] Acosta J., Penela D., Andreu D. (2018). Multielectrode vs. point-by-point mapping for ventricular tachycardia substrate ablation: a randomized study. *EP Europace*.

[22] Di Biase L., Santangeli P., Burkhardt D. J. (2012). Endo-epicardial homogenization of the scar versus limited substrate ablation for the treatment of electrical storms in patients with ischemic cardiomyopathy. *Journal of the American College of Cardiology*.

[23] Tzou W. S., Frankel D. S., Hegeman T. (2015). Core isolation of critical arrhythmia elements for treatment of multiple scar-based ventricular tachycardias. *Circulation: Arrhythmia and Electrophysiology*.

[24] Bogun F., Good E., Reich S. (2006). Isolated potentials during sinus rhythm and pace-mapping within scars as guides for ablation of post-infarction ventricular tachycardia. *Journal of the American College of Cardiology*.

[25] Jaïs P., Maury P., Khairy P. (2012). Elimination of local abnormal ventricular activities : a new end point for substrate modification in patients with scar-related ventricular tachycardia. *Circulation*.

[26] Blomström-Lundqvist C., Auricchio A., Brugada J. (2013). The use of imaging for electrophysiological and devices procedures: a report from the first European heart rhythm association policy conference, jointly organized with the European association of cardiovascular imaging (EACVI), the council of cardiovascular imaging and the European society of cardiac radiology. *Europace*.

[27] Martin R., Maury P., Bisceglia C. (2018). Characteristics of scar-related ventricular tachycardia circuits using ultra-high-density mapping. *Circulation: Arrhythmia and Electrophysiology*.

