# Supplementary material for: Automatic Detection of Slow Conducting Channels during Substrate Ablation of Scar-Related Ventricular Arrhythmias
Source: J Interv Cardiol. 2020 May 29;2020:4386841. doi: 10.1155/2020/4386841 (PMC7275947; doi:10.1155/2020/4386841)
Supplement: Supplementary Materials — Supplementary Table 1: Lin's concordance correlation factor on the number of SCC entrances identified between mapping modalities (rows against columns) from the analysis of the colour-coded maps. Supplementary Table 2: Lin's concordance correlation factor on the number of SCC entrances identified between EAM standard maps and SCC-Maps from the analysis of EGM-DC and identification of f-EGMs. [file 4386841.f1.pdf]

SUPPLEMENTARY MATERIALS

SUPPLEMENTARY TABLES

Supplementary table 1. Lin's Concordance Correlation factor on the number of SCC entrances identified between mapping modalities (rows against columns) from the analysis of the colour-coded maps.

		vs. EAM screening	vs .SCC-Maps	vs. Ce-CMR PSI Maps
**EAM standard**	**Entire population (n = 20)**	0.323*	0.227*	N/A
	**Ischemic (n = 10)**	0.366*	0.156*	0.212*
	**ARVD/C (n = 10)**	0.047*	0.023*	N/A
**EAM screening**	**Entire population (n = 20)**	-	0.665*	N/A
	**Ischemic (n = 10)**	-	0.528*	0.679*
	**ARVD/C (n = 10)**	-	0.877*	N/A

**SCC-Maps**	**Entire population (n = 20)**	-	-	N/A
	**Ischemic (n = 10)**	-	-	0.628*
	**ARVD/C (n = 10)**	-	-	N/A

**P-value < 0.01.*
ARVD/C: Arrhythmogenic right ventricular dysplasia/cardiomyopathy; Ce-CMR: Contrast-enhanced cardiac magnetic resonance; EAM: Electroanatomical Mapping; N/A: Not applicable; PSI: Pixel signal intensity; SCC: Slow conducting channel.

Supplementary table 2. Lin's Concordance Correlation factor on the number of SCC entrances identified between EAM standard maps and SCC-Maps from the analysis of EGM-DC and identification of f-EGMs.

	**Lin's Concordance Correlation factor**
**Entire population (n = 20)**	0.918*
**Ischemic (n = 10)**	0.871*
**ARVD/C (n = 10)**	0.936*

**P-value < 0.01.*

ARVD/C: Arrhythmogenic right ventricular dysplasia/cardiomyopathy; EAM: Electroanatomical Mapping; EGM-DC Electrograms with delayed components; f-EGM: fused electrograms; SCC: Slow conducting channel
